# Facilitators and barriers of long-term exercise adherence in healthcare workers formerly suffering from post-COVID-19 syndrome: A qualitative 1-year follow-up and quantitative pilot study of the COFIT trial

**DOI:** 10.1007/s00508-024-02446-x

**Published:** 2024-10-04

**Authors:** Timothy Hasenöhrl, Beate Scharer, Margarete Steiner, Jim Schmeckenbecher, Galateja Jordakieva, Richard Crevenna

**Affiliations:** 1 Department of Physical Medicine, Rehabilitation and Occupational Medicine, Medical University of Vienna, Waehringer Guertel 18–20, 1090 Vienna, Austria; 2 Competence Center for Occupational Safety and Health Maintenance (CCAG) of the General Hospital of Vienna and the Medical University of Vienna, Vienna, Austria

**Keywords:** Long COVID, COVID-19 long haulers, Training, Compliance, Rehabilitation

## Abstract

**Background:**

Early exercise intervention studies showed promising positive effects of physical exercising on post-COVID-19 symptoms; however, little is known about long-term training adherence and what influences it.

**Material and methods:**

Semi-structured interviews were conducted with 17 participants of the 8‑week original exercise intervention study. Facilitators and barriers were identified via thematic analysis and compared between those participants who continued their regular exercise behavior (continuous exercise group, CEG, *n* = 7) and those who stopped exercising (discontinuous exercise group, DEG, *n* = 10). Physical performance parameters and questionnaires regarding psychological health dimensions and work ability were assessed analogously to the original study.

**Results:**

Qualitative analysis showed that two of the top three facilitators, (improving physical and mental health, sport has high priority) were the same in both groups. The respective third of the top three facilitators was (re)build physical and cognitive performance in the CEG and training in the group in the DEG. The top three barriers (exhaustion, sport has little priority, procrastination) were not only the same in both groups but also in the same order.

**Conclusion:**

The strongest post-COVID-19 associated facilitator for long-term exercise adherence is when the need for further reconditioning is felt. The strongest post-COVID-19 associated barrier is exhaustion. Availability of exercising in a group is a key factor in increasing long-term exercise adherence.

## Introduction

As of early September 2023, there were 770 million confirmed cases of COVID-19, including close to 7 million deaths and therefore approximately 760 million survivors globally [1]. Although most infected people fully recovered from their initial infection, a substantial proportion of COVID-19 survivors suffered and continued to suffer from a variety of long-term symptoms. Estimations of incidence of ongoing symptoms ranged between 50% and 70% in formerly hospitalized patients and between 10% and 30% in non-hospitalized patients [2, 3]. The COVID-19 vaccines appear to have had an impact on the incidence of post-COVID-19 symptoms, as it decreased to 10–12% in vaccinated COVID-19 survivors [4, 5]. Nevertheless, even the most conservative estimates pose post-COVID-19 syndrome as a significant healthcare challenge in the next few years, with at least 65 million COVID-19 survivors expected to suffer from post-COVID-19 symptoms [6].

The post-COVID-19 syndrome is manifested by a variety of symptoms such as fatigue, dyspnea, cough, pain, insomnia, headache, loss of taste and smell [7] and cognitive dysfunction might either persist from the initial illness or develop only after recovery [8]. These symptoms can come and go or relapse over time and most notably, they can negatively affect a person’s physical and cognitive function, health-related quality of life, and participation in society [9]. Given the high variability of post-COVID-19 syndrome, it was hypothesized even early after its discovery that tailored and supervised physical training could be an effective systemic therapy suitable for a wide range of different cases and symptoms [7].

This early hypothesis has now been supported by initial findings from exercise intervention studies indicating that exercise training in COVID-19 survivors suffering from post-COVID-19 syndrome lead to improvements in physical fitness and mental health and to a reduction in COVID-associated functional impairments and fatigue [10, 11].

Similar effects were shown by our research group in an exercise intervention study with healthcare workers suffering from post-COVID-19 syndrome [12]. In this study, 32 healthcare workers suffering from either mild or severe post-COVID-19 related symptoms underwent an 8‑week exercise protocol consisting of a supervised progressive resistance exercise group program together with individualized aerobic exercise recommendations based on ventilatory thresholds. In this study, we were able to show that this kind of exercise program led to improvements in physical fitness and correlations with improvements in mental health and work ability [12]. The aim of the original project was to find out if physical training might be a potent countermeasure against post-COVID-19 syndrome and if there might be a difference in the trainability of COVID-19 survivors suffering from severe post-COVID-19 syndrome compared to those with only minor post-COVID-19 symptoms. The results showed that both higher endurance capacity as well as higher muscle strength were related to less fatigue as well as better functional ability and improved working ability. Moreover, the results of the 30 s sit-to-stand test (30secSTS) correlated with an improvement in fatigue levels as well as the psychological outcomes stress, depression, and anxiety. In addition, we showed that post-COVID-19 severity did not affect trainability [12].

This current article presents the results of the 1‑year follow-up of this trial. The aim of this 1‑year follow-up (1yrFU) was to identify facilitators and barriers of long-term therapy adherence to the exercise intervention. In this respect, in the main analytical part of this study, semi-structured interviews were performed with the participants of the initial project. Additionally, in the secondary explorative pilot study part, physical function and psychological health outcomes were assessed as a measure of the development of the long-term physical fitness and mental health.

## Methods

### Participants

A total of 29 healthcare workers from the original study completed the interventions and assessments [12]. These former participants were contacted and invited to participate in the 1‑year follow-up study. Of the participants 1 was known to no longer work at the hospital and could not be contacted and 11 former participants were not interested and did not respond. This left 17 participants who were willing to take part in the 1‑year follow-up.

The present study has a mixed methods design with a qualitative part and a quantitative part. The primary aim, the identification of long-term therapy adherence, was approached utilizing qualitative research methods and a combination of deductive and inductive category development. The semi-structured interviews were divided into two sections depending on therapy adherence (further training yes/no) and eight main categories were defined beforehand, namely therapy adherence, method of implementation, motivational factors, personal training history, general well-being, quality of sleep, cognitive abilities, and social relationships.

The interviews were saved via audio recording for later transcription and analysis. Category formation according to Mayring (2014) [13] was chosen as a suitable procedure for analyzing recorded data. As a unit of analysis, we defined each statement about motivation factors to continue the training or about factors that hinder it, why it was not possible or only possible with difficulty. Statements about motivating and hindering factors were paraphrased with the aim of deleting excess material from a statement. All paraphrases below the level of abstraction were generalized, paraphrases above the level of abstraction were initially left as they were. The generalization resulted in passages with the same content. Paraphrases with the same meaning were deleted. In the second reduction, the categories were further reduced by presenting the statements across all cases. The summary category system was checked again on the source material and whether categories match what people actually said. In the end, a category system was formed from the comprehensive interview transcript with the aim of relating the statements to one another and being able to use categories to generate a hypothesis to answer the research question. For data management and analysis, we used Atlas.ti (version 9.1.7, ATLAS.ti Scientific Software Development GmbH, Berlin, Germany).

As the secondary aim of this study, participants of the original study were also invited to perform a 1-year follow-up assessment of the functional tests and questionnaires. This included tests of physical performance (30 s sit-to-stand test, 6‑minutes walk test, handgrip strength test), body composition (bioimpedance analysis), work ability (Work Ability Index), post-COVID functional status (Post-Covid Functional Scale), fatigue (Brief Fatigue Inventory), as well as questionnaires for psychological health dimensions like anxiety (Generalized Anxiety Disorder 7), stress (Perceived Stress Scale-10), depression (Patient Health Questionaire-9), resilience (Brief Resilience Scale), and sleep (Insomnia Severity Index).

Depending on their long-term exercise adherence, participants were stratified into two groups. Those who continued with their physical training and practiced endurance as well as resistance training at least twice a week over the past year were allocated to the continuous exercise group (CEG). Those who discontinued their exercise regime were allocated to the discontinuous exercise group (DEG).

Quantitative results were analyzed within the frame of being secondary pilot data within this part of the project. To reflect the change of physical performance over the whole study duration since the start of the original exercise intervention at baseline (BL), the CEG versus DEG mean values were plotted in graphs. Following this, the performance outcome parameters were correlated with the psychological health dimensions at the 1yrFU via Spearman rank correlation. To test potential differences between those in the CEG and DEG group, *t*-tests or a Wilcoxon test were used. Comparison between the end of the original exercise intervention, the 8‑week follow-up (8wkFU), and the 1yrFU were calculated. The rest of the parameters, which did not show substantial changes over the 8 weeks of exercise intervention within the original study, were analyzed regarding their differences between the BL and the 1yrFU as well as between the 8wkFU and the 1yrFU with the aim of potentially identifying long-term effects.

The study protocol was approved by the ethics committee of the Medical University of Vienna, Austria (EK1181/2021) and was performed in accordance with the ethical standards laid down in the 1964 Declaration of Helsinki. All participants gave their informed consent prior to their inclusion in the study.

The present analyses took place within the scope of the larger project COFIT, which focusses on the effects of exercise interventions on various dimensions of the post-COVID syndrome (NCT04841759 at ClinicalTrials.gov).

## Results

Those participants of the original interventional study who completed all former study assessments (*n* = 29 of 32) were invited to participate in the 1yrFU. The interviews took place between May and June 2022. A total of 17 participants provided answers to the open-ended questions of the conducted semi-structured interviews. When analyzing the transcripts, no additional categories were deductively developed, so the eight main categories developed inductively covered all relevant information and therefore remained as they were. After 14 interviews, data saturation regarding barriers and facilitators was achieved.

Women represented 76.5% of the sample and men 23.5%. Of the 17 participants who participated in the 1yrFU, 4 contracted COVID-19 again, which corresponds to 23.5%.

At the 1yrFU the average age of the sample was 49.9 ± 8.9 years and did not differ between group allocation. The CEG consisted of 7 people (41.2%) and the DEG of 10 participants (58.8%). Detailed information about the characteristics of the participants at the 1yrFU are depicted in Table 1. No significant group differences between CEG and DEG were noticed at the 1yrFU (Table 1).

**Table 1:** Participant 1‑year follow-up characteristics differentiating between group allocation

	Total	CEG	DEG
*n*	17	7	10
Age (years) Mean ± SD	49.9 ± 8.9	49.4 ± 3.9	50.2 ± 11.4
1yrFU group difference (*p*-value)	–	*p* = 0.846	
Height (cm) Mean ± SD	171.8 ± 6.2	171.1 ± 7.3	172.3 ± 5.7
1yrFU group difference (*p*-value)	–	*p* = 0.719	
Weight (kg) Mean ± SD	75.4 ± 19.1	68.4 ± 11.2	80.3 ± 22.4
1yrFU group difference (*p*-value)	–	*p* = 0.172	
Fat % (%) Mean ± SD	28.1 ± 7.6	26.7 ± 5.8	29.0 ± 8.8
1yrFU group difference (*p*-value)	–	*p* = 0.558	
Fat Mass (kg) Mean ± SD	22.0 ± 10.8	18.3 ± 4.5	24.6 ± 13.2
1yrFU group difference (*p*-value)	–	*p* = 0.187	
Fat free mass (kg) Mean ± SD	53.4 ± 11.0	50.1 ± 9.7	55.7 ± 11.7
1yrFU group difference (*p*-value)	–	*p* = 0.321	
Muscle mass (kg) Mean ± SD	50.7 ± 10.5	47.6 ± 9.2	52.9 ± 11.2
1yrFU group difference (*p*-value)	–	*p* = 0.321	
BMI (kg/m^2^) Mean ± SD	25.3 ± 5.5	23.3 ± 2.6	26.8 ± 6.6
1yrFU group difference (*p*-value)	–	*p* = 0.153	

*1yrFU* 1-year follow-up, *BMI* body mass index, *CEG* continuous exercise group, *DEG* discontinuous exercise group, *SD* standard deviation

Facilitators, including how often a statement related to a specific category was mentioned separately during the interviews are depicted in Table 2 under the term frequency. Improving physical and mental health was named most frequently as a motivating factor for physical activity with 19 mentions. Respondents in this category reported benefiting from sports or exercise both physically and mentally:

“Sport is not only good for me physically, but it is also extremely good for my mental health” (Irene, 53, F, CEG).

**Table 2:** The 10 most frequently mentioned facilitators and barriers across the entire study population and depending on group allocation CEG and DEG

Facilitators	Total frequency	Frequency in the CEG	Ranked in CEG	Frequency in the DEG	Ranked in DEG
Improving physical and mental health	19	**10**	**3**	**9**	**2**
Sport has high priority	18	**11**	**1**	**7**	**3**
(Re)build physical and cognitive performance	15	**11**	**1**	4	6
Training in the group	13	3	11	**10**	**1**
Better sleep through exercise	12	6	6	6	4
Activity tracking	10	6	6	4	6
Guided training	10	6	6	4	6
Training protects against injuries and pain	9	7	4	2	12
Knowledge about one’s own performance or body composition	9	7	4	2	12
Summer nice weather and brightness	8	3	11	5	5
*Barriers*					
Exhaustion	21	**7**	**1**	**14**	**1**
Sport has little priority	17	**5**	**2**	**12**	**2**
Procrastination	12	**4**	**3**	**8**	**3**
Covid-19 measures and risk of infection	8	2	5	6	4
(Certain) sport is no fun	8	2	5	6	4
Winter and bad weather/darkness	6	2	5	4	6
Having to do sports alone	4	–	–	4	6
Family and domestic commitments	4	1	8	3	8
No fitness center nearby	3	1	8	2	11
Illness/pain	3	–	–	3	8

*CEG* continuous exercise group, *DEG* discontinuous exercise group

Bold numbers highlight the frequency and the ranking of the top-three ranked facilitators and barriers in the respective group

Sport has high priority was named 18 times. It was described by the participants in this category that performing sport had great importance in their lives and was therefore prioritized over other things.

“Sport is a fixed part of my life” (Elisabeth, 47, F, CEG)

The category (re)build physical and cognitive performance was mentioned 15 times. A measurable or noticeable increase in physical and mental endurance was described by participants as motivating:

“It motivated me to continue, because I realized how successful I had been” (Anja, 50, F, CEG)

Training in the group with 13 mentions was also of high priority regarding motivating factors for physical activity. The participants found it a motivating factor not to pursue a sporting activity alone, but together with other people, as this enabled them to motivate each other.

“Yes, as I said, it’s motivating that there are two of us” (Claudia, 55, F, CEG)

Barriers, including how often a statement related to a specific category was mentioned during the interviews are depicted in Table 2 under the term frequency.

Exhaustion was mentioned 21 times as the primary barrier. This included exhaustion both in terms of post-COVID-19-related symptoms as well as the exhaustion due to numerous day to day responsibilities (job, family).

“When you come home from work exhausted, you don’t have enough energy for a workout, which is demotivating” (Renate, 42, F, CEG).

Sport has little priority (mentioned 17 times). This category included statements that were primarily based on a lack of time resources to engage in sporting activities.

“Yes, (… I try to have time for sports, but only …) depending on how it goes, how it works out in terms of time, with family and work and yes appointments” (Anton, 53, M, CEG)

Procrastination (mentioned 12 times). Participants described that they often lacked the inner will to motivate themselves to exercise.

“I would not have been able to overcome my weaker self” (Alois, 42, M, DEG)

COVID-19 measures and risk of infection (mentioned 8 times). On the one hand, due to the lockdowns COVID-19 severely restricted the range of sports, for example in a fitness center. On the other hand, participants also described the fear of contracting COVID-19 when exercising in a separate room with other people.

“And I also used to have the fitness center before COVID. But then I did not want that during the pandemic” (Ursula, 63, F, DEG).

To gain a more detailed insight into the facilitators and barriers to physical activity, we separated participants who reported continuation of their regular aerobic and resistance exercising from those who discontinued exercising in the past year.

When comparing the two subgroups in terms of facilitators for exercising, we found that participants in the CEG mentioned 14.6 motivational factors per participant, while in the DEG only 7.7 motivational factors were mentioned per participant. When comparing the two subgroups in terms of barriers for exercising, we found that participants in the CEG mentioned 3.9 hindering factors per participant, while in the DEG 6.8 hindering factors were mentioned per participant. The absolute number of facilitator mentions was higher in the CEG compared to the DEG (102 vs. 77). On the other hand, putting the categories in order shows that the top three facilitator categories were similar for each group. While the top three facilitators in the CEG were (re)build physical and cognitive performance, sport has a high priority, and improving physical and mental health, the top three in the DEG were training in the group, improving physical and mental health, and sport has a high priority. This means that two of the top three facilitators were the same in both groups. The groups differentiated with respect to the factors (re)build physical and cognitive performance, which was substantially higher prioritized in the CEG (1.6 mentions/participant vs. 0.4 mentions/participant) and training in a group, which was substantially higher prioritized in the DEG (1 mention/participant vs. 0.4 mentions/participant).

“I knew I had to continue exercising to not get long-COVID again” (Carla, 50, F, CEG).

“So maybe the motivation to be in a group, that you have to or should come regularly or so this group feeling” (…) is beneficial for practicing regularly (Stefanie, 30, F, DEG).

In absolute numbers, barriers were mentioned more often in the DEG compared to the CEG (68 vs. 27); however, the order of the categories revealed that the top three barriers, exhaustion, sport has little priority and procrastination, were not only the same in both groups but also appeared in the same order. Exhaustion, as the primary barrier in both groups, was mentioned twice as often in the DEG than in the CEG group (14 vs. 7 mentions). Participants referred to actual physical exhaustion that prevented them from engaging in physical activity, and which affected participants in the DEG group more often.

“Sometimes nothing worked at all, where I had the feeling that every movement was torture. Pain in the limbs, exhaustion, (…), where everything was an overwhelming effort and nothing worked anymore and the shortness of breath was also stronger again” (Laura, 59, F, DEG).

Furthermore, exhaustion was also mentioned related to the fear of being re-infected with COVID-19. This was mentioned six times in the DEG, while the CEG it was mentioned only twice. Participants in the DEG also mentioned more often than those in the CEG that other priorities prevented them from exercising more often (sport has little priority, 12 mentions in the DEG vs. 5 mentions in the CEG).

“So there’s energy for things like that, I just don’t have the time to juggle it all” (Erwin, 49, M, DEG).

Procrastination was mentioned twice as often in the DEG compared to the CEG (8 vs. 4 mentions).

“These are my inner demons, as they say. They are just very strong with me” (Elvira, 64, F, DEG)

Nevertheless, despite the differences in absolute numbers, the order of the top three barriers were the same in both groups.

**Fig. 1:**
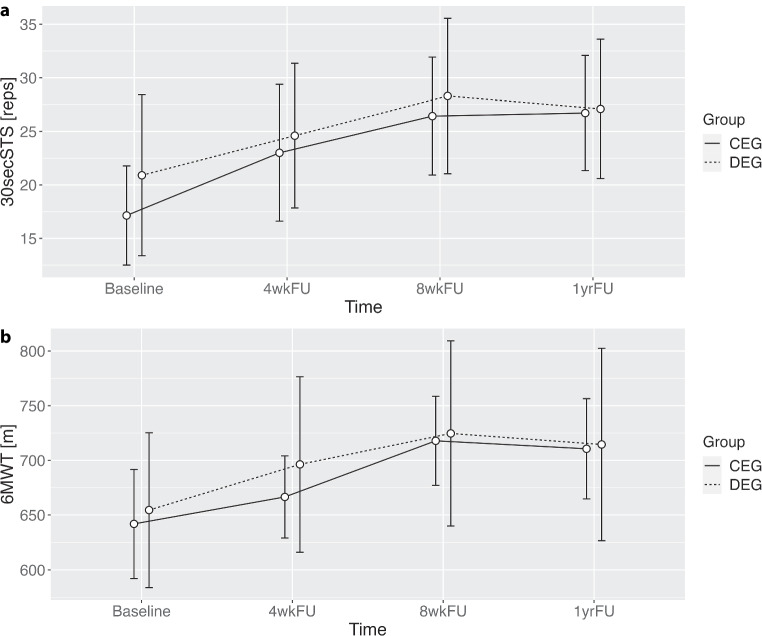
Development of performance parameters (**a** 30secSTS and **b** 6 MWT) over the duration of the whole study including the initial supervised exercise intervention phase. *4wkFU* 4-week follow-up, *8wkFU* 8-week follow-up, *1yrFU* 1-year follow-up, *CEG* continuous exercise group, *DEG* discontinuous exercise group, *30secSTS* 30s sit-to-stand test, 6 *MWT* 6-min walk test, *reps* repetitions

The physical performance parameters 30secSTS and 6 MWT showed a visible increase during the exercise intervention phase of the original study ([12]; Fig. 1a, b). The group comparison CEG versus DEG, which gets relevant only after the 8‑week follow-up (8wkFU) of the original study [12], showed a slight increase in the mean 30secSTS performance between the 8wkFU and the 1yrFU for the CEG, while the mean number of repetitions decreased in the DEG at the same time (Fig. 1a); however, inferential tests showed that this difference was not significant (*p* = 0.29). Similar to the 30secSTS results, the 6 MWT also showed a performance increase between BL and 8wkFU during the original intervention period [12]; however, in the long-term between the 8wkFU and the 1yrFU, both the CEG and the DEG decreased their performance similarly (Fig. 1b). Unsurprisingly, the mean differences between the groups did not differ significantly in the 6 MWT (*p* = 0.82).

Further quantitative outcome parameters are shown in Table 3. A significant increase in dominant handgrip strength between BL and the 1yrFU (t(6.52) = −2.42, *p* = 0.049) was shown. (Table 3) Moreover, a significant change in the PCFS between BL and 1yrFU (z = 2.18, *p* = 0.048) with lower scores in the CEG compared to the DEG was shown (Table 3).

**Table 3:** Change of outcome parameters over time points (BL, 8wkFU and 1yrFU) between CEG and DEG

Variable	Test	Statistic (t or z value)	Df (t-test only)	*p*
*Change of CEG and DEG between baseline and 1yrFU*				
HGS (dominant)	t‑test	−2.42	6.52	0.049*
HGS (non-dominant)	t‑test	−2.10	7.60	0.071
Post-COVID Functional scale	u‑test	2.18	–	0.048*
Fat (%)	u‑test	0.88	–	0.42
Fat, mass (kg)	u‑test	1.46	–	0.16
Fat free mass	t‑test	1.17	13.17	0.26
Muscle mass	t‑test	1.18	13.08	0.26
BMI	u‑test	1.32	–	0.20
Bodyweight	t‑test	1.55	10.37	0.15
*Change of CEG and DEG between 8wkFU and 1yrFU*				
HGS (dominant)	t‑test	0.36	8.86	0.73
HGS (non-dominant)	t‑test	−0.98	6.57	0.36
Post-COVID Functional scale	u‑test	1.64	–	0.19
Fat (%)	t‑test	1.31	8.44	0.23
Fat, mass (kg)	t‑test	1.70	7.88	0.13
Fat free mass	t‑test	1.44	13.92	0.1716
Muscle mass	t‑test	1.42	13.93	0.1771
BMI	t‑test	1.85	9.55	0.0948
Bodyweight	t‑test	1.96	10.14	0.07833

*BMI* body mass index, *HGS* handgrip strength, *CEG* continuous exercise group, *DEG* discontinuous exercise group, *BL* baseline, *8wkFU* 8-week follow-up, *1yrFU* 1-year follow-up

Between the 8wkFU and the 1yrFU no significant change was observed in any of the parameters, however, in the body mass index (BMI) there was a trend (t(9.55) = 1.85, *p* = 0.095) towards lower values in the CEG.

**Fig. 2:**
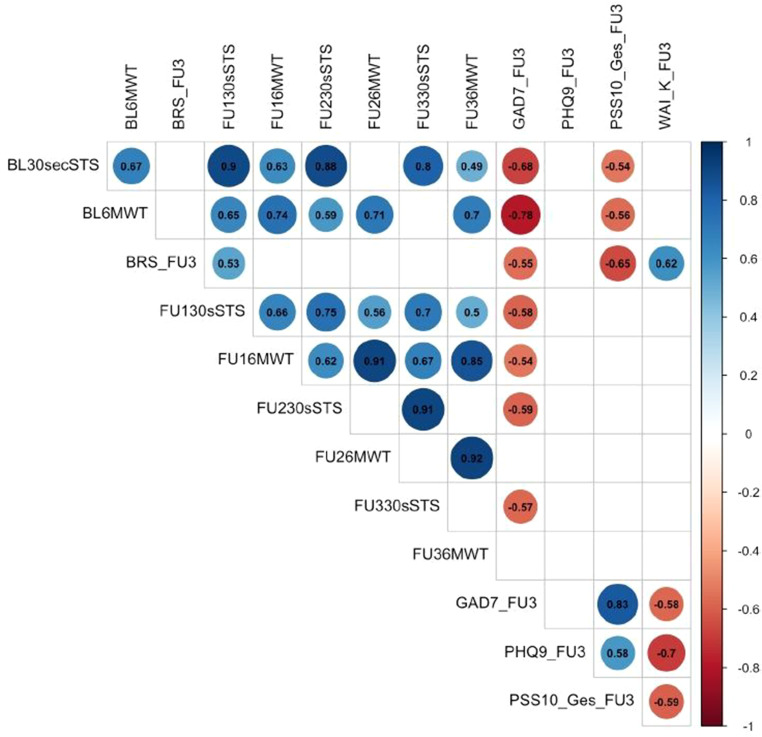
Spearman correlation between performance outcomes and psychological health outcomes. *BL *baseline, *FU1 *4-week follow up, *FU2 *8-week follow-up, *FU3 *1-year follow-up, *30secSTS *30-seconds sit-to-stand test, *6MWT *6-minute walk test, *BRS *brief resilience scale, *GAD-7* generalized anxiety disorder-7, *PHQ-9* patient health questionnaire-9, *PSS-10-Ges* perceived stress scale-10 total score, *WAI-K *work ability index short form

Spearman correlation analysis was calculated to analyze the relationship between performance outcomes and psychological health outcomes. At the 1yrFU, the only psychological health outcome which significantly correlated with a performance parameter at the same time point was anxiety (GAD-7) which showed a significant inverse correlation with the 30secSTS (ρ = −0.57) (Fig. 2).

## Discussion

Our qualitative study results showed that independent from long-term exercise adherence, the majority of facilitators and barriers did not differ substantially in post-COVID survivors. Of the top three facilitator categories two, namely, improving physical and mental health and sport has a high priority, were the same in both the CEG and the DEG. The third facilitator in the CEG was (re)build physical and cognitive performance and in the DEG it was training in the group.

On the other hand, the top three barriers exhaustion, sport has little priority, and procrastination were not only the same but they were even mentioned in the same order in both groups. The only difference between the groups was the absolute number of mentions of each category.

The current study is, to our knowledge, the first analysis of barriers and facilitators to long-term exercise adherence in post-COVID survivors. We identified sport has a high priority as one of the most important facilitators in our study sample. The high priority in our participants was shown by organizing the day around physical activity, e.g., by setting of a protected time. Moreover, participants who had a high priority for sport mentioned that they were particularly flexible in facilitating their exercise sessions (e.g., if they did not have enough time for practicing in the gym, they were practicing at home) and that they included physical activity in their vacations. This finding is novel for post-COVID survivors. It could be argued that healthcare workers who are comparably well-educated people with a higher socioeconomic status, have a better developed health behavior [14]; however, the exact opposite and one of the top ranked barriers for exercising in our study was sport has little priority. Therefore, socioeconomic status cannot explain why the priority of sport was both a facilitator and a barrier in our study population.

Participants of both the CEG and the DEG mentioned that improving physical and mental health was a facilitator to physical activity. This goes in line with several findings from previous studies. Gilbert et al. (2019) reported physical, social and mental well-being as primary sources of motivation for physical activity in people from rural communities [15]. Roche et al. (2022) on the other hand, mentioned that during the pandemic many participants felt that physical activity had a positive effect on their mental health and that this was one of the primary facilitators for most of the demographic groups [16].

Only the third of the top three facilitators differed between the CEG and the DEG, and interestingly it was the factor mentioned most frequently in the respective groups. In the CEG,(re)build physical and cognitive performance was one of the most important facilitators for exercising. A closer look at the quantitative results revealed that this facilitator was mentioned slightly more often from participants who suffered from either a physical or mental impairment at the end of the supervised exercise intervention study, hence the beginning of the long-term follow-up phase. Considering the fact that this facilitator can be viewed as a factor related to post-COVID-19 symptomology, this would mean that these participants might have felt increased need for further physical and mental improvement compared to those less affected and were therefore more likely to stay continuously physically active. We would therefore suggest that the facilitator (re)build physical and cognitive performance can be considered thematically close to the commonly reported facilitator improving physical and mental health [16, 17] but with a specific post-COVID-19 rehabilitation or reconditioning aspect.

In the DEG training in the group was the unique facilitator for exercising. Group exercising as a facilitator has been reported several times in various patient populations [18, 19]. Practicing physical activity in a group is strongly connected to social support and is a consistent determinant of physical activity [20]. A qualitative study with patients suffering from low back pain gave more insights by reporting that it is more motivating to train in a group because the risk of finding excuses and postponing physical activity decreases [21]. With respect to our study this means that considering the factthat during the COVID-19 pandemic participation in group exercising was limited, it is just logical that those participants who prioritized training in the group were more likely to discontinue their physical exercising (DEG).

It was not just the absence of facilitators which prevented the study participants of the DEG from continuously exercising, both groups were confronted with the same barriers as well. The primary barrier for the whole study sample as well as both subgroups was exhaustion. Low energy is described as a factor which decreased participants ability to engage in physical activity and exercise [22] and is therefore known to being a huge barrier to physical activity [18, 20, 23, 24]. In our study, exhaustion was mentioned as a barrier by twice as many participants in the DEG than in the CEG (14 vs. 7) and was particularly related to post-COVID-19 symptoms.

On the other hand, the barrier procrastination, which was mentioned by 8 participants in the DEG and by 4 in the CEG, was not related to post-COVID-19 symptoms in our study sample. Procrastination has been known to be a factor contributing to intention-behavior gaps [25]. Participants reported time constraints and the lack of self-discipline as barriers for participating in physical activities. Similar factors have been reported in participants suffering from mood and/or anxiety problems [26]. Although this study took place during a still-active phase of the COVID-19 pandemic and after a series of lockdowns, participants in this study no longer exhibited increased anxiety levels from the end of the exercise intervention phase, therefore, the reported procrastination was assumed not to be associated with anxiety or mood issues; however, less than 150 min of physical activity per week has been shown to be associated with less positive perceptions of quality of life related to physical and mental health and that this perception is in turn related to higher levels of procrastination [27]. In addition, there is significant variability in self-reported exercise procrastination and exercise procrastination is associated with lower overall physical activity, even after accounting for intention and overall delay [28].

The COVID-19 restrictions and the increased risk of (re)infection, although they were mentioned by the participants as barriers to physical activity, played only a minor part in both groups, compared to other barriers. This is particularly interesting as all of the participants somehow had been affected by post-COVID syndrome in the first place. This could be explained by the availability of various COVID-19 vaccines to healthcare workers at the time of the interviews and the consequent loss of threat levels [29].

Of the top three facilitators and barriers in the CEG and the DEG, the majority of factors, namely improving physical and mental health, sport has high/little priority, and procrastination was unrelated to post-COVID-19 symptomology. For patients primarily reporting these factors it appears that the factors for (dis)continuing individual exercise behavior were the same as in the normal healthy population. This estimation is supported by the fact that the physical performance levels as well as the psychological domains did not differ between the groups at the start of the long-term follow-up period, the 8wkFU. Moreover, at the 1yrFU these outcomes showed to be still substantially elevated compared to the baseline levels and they were consistently within normal ranges. This would suggest that for the majority of the included participants, further reconditioning or rehabilitation was no longer necessary. Therefore, the individual need for further exercising to improve fitness and the associated health dimensions even further was no longer present, so ordinary facilitators and barriers apart from post-COVID-associated motivators won influence again. This means that in contrast to our a priori assessment of this long-term follow-up study, in which we considered the 8‑week exercise intervention to be rather short in order to produce longer lasting results, 8 weeks of structured and supervised combined aerobic exercise and resistance training appears to be sufficient to replace initial post-COVID-19 facilitators and barriers with common individual facilitators and barriers to long-term exercise adherence in the majority of patients.

However, there are still patients who experience post-COVID-19-related facilitators and barriers. The primary facilitator in the CEG, (re)build physical and cognitive performance, and the primary barrier in both groups, exhaustion, were factors directly related to post-COVID-19 symptoms. The primary facilitator in DEG, training in the group, may have had something to do with living conditions during the pandemic but was actually independent of post-COVID symptoms.

A limitation of this study is that the sample size was rather small. As the primary outcomes of this 1yrFU were predominantly qualitative, this is not of great importance, particularly as data saturation was reached; however, even though a response rate of nearly 60% in a long-term follow-up study is respectable, the rather small sample size remains a limitation. Moreover, our results may only be partially representative of the general population because our study sample consisted of healthcare workers. Healthcare workers have a better medical education than the general population and showed a more positive attitude towards COVID-19 vaccines [29]. Moreover, they never experienced workplace restrictions and lockdowns similar to the general population. During the pandemic they were even more challenged in their daily work and many of them have been actively in the service despite suffering from post-COVID-19-related symptoms. On the other hand, this limitation is a particular strength of this study as we gained detailed insights into this patient population, which has shown its exceptional importance over the years of the pandemic.

Due to the large prevalence of long-lasting post-COVID-19 syndromes in healthcare workers [30] there is still a strong demand for further research of how this population can be best supported during their rehabilitation. Based on the results of the original study [12] and the current follow-up study, it would make sense to compare different exercise intervention durations with the aim of identifying how long additional positive effects from exercise on post-COVID-19 symptoms can be achieved beyond 8 weeks of supervised exercising.

## Conclusion

If after 8 weeks of targeted, supervised training non-hospitalized post-COVID-19 patients, who initially never experienced worse than moderate post-COVID-19 symptoms (PCFS ≤ 3), still feel that the original physical and cognitive performance has not yet been reached, there is a high probability that they will continue training despite these symptoms; however, if patients are suffering primarily from exhaustion, offering participation in group exercise should increase exercise adherence. In all other cases, most post-COVID-19 symptoms have improved to a point where the need for post-COVID-19 rehabilitation is no longer perceived and other, normal factors now determine whether exercising continues or not.

## Data Availability

The data supporting both the qualitative and quantitative findings of this study are available from the corresponding author upon reasonable request.
