# Correction to: Facilitators and barriers of long-term exercise adherence in healthcare workers formerly suffering from post-COVID-19 syndrome

**DOI:** 10.1007/s00508-024-02473-8

**Published:** 2024-11-05

**Authors:** Timothy Hasenöhrl, Beate Scharer, Margarete Steiner, Jim Schmeckenbecher, Galateja Jordakieva, Richard Crevenna

**Affiliations:** 1 Department of Physical Medicine, Rehabilitation and Occupational Medicine, Medical University of Vienna, Waehringer Guertel 18–20, 1090 Vienna, Austria; 2 Competence Center for Occupational Safety and Health Maintenance (CCAG) of the General Hospital of Vienna and the Medical University of Vienna, Vienna, Austria

**Correction to:**

**Wien Klin Wochenschr 2024**

10.1007/s00508-024-02446-x

In this article the statement in the Funding information section was incorrectly given as: “Funding Open access funding provided by Medical University of Vienna.” and should have read: “Funding Open access funding provided by Medical University of Vienna. The project was funded by the Medizinisch-Wissenschaftlichen Fonds der Stadt Wien.”

The original article has been corrected.

